# Anticipatory Anxiety, Familiarization, and Performance: Finding the Sweet Spot to Optimize High-Quality Data Collection and Minimize Subject Burden

**DOI:** 10.3390/ejihpe12090094

**Published:** 2022-09-09

**Authors:** Aspen E. Streetman, Aidan K. Lewis, Elizabeth L. Rogers, Katie M. Heinrich, Justin A. DeBlauw

**Affiliations:** 1 Department of Kinesiology, Kansas State University, Manhattan, KS 66506, USA; 2 Department of Health and Human Physiological Sciences, Skidmore College, Saratoga Springs, NY 12866, USA

**Keywords:** research protocols, data collection, best practices for research, HIFT, sports psychology

## Abstract

Accurate baseline data are essential for researchers to determine an intervention’s effects yet may be affected by anticipatory anxiety and assessment familiarity. Familiarization sessions help establish accurate baseline data. High-intensity functional training (HIFT) elicits performance outcomes based on constantly varied workouts. It is unclear how familiarization affects anticipatory anxiety and workout performance among HIFT novices. Familiarization was hypothesized to decrease anxiety and improve workout performance. Sixteen college-aged subjects (62.5% women, 20.2 ± 1.14 years) completed one introductory and four sessions of the same workout. All subjects were recreationally trained with no HIFT experience. State and trait anxiety were assessed at the first session. During the workout sessions, state anxiety (SQALS) was assessed upon arrival at the gym (SQALS 1), after learning the workout protocol (SQALS 2), and when the workout concluded (SQALS 3). A significant main effect of the number of previous sessions on workout performance was observed (*p* = 0.011). A repeated-measures ANOVA showed a main effect of time on SQALS 1 (*p* < 0.001), SQALS 2 (*p* < 0.001), and SQALS 3 (*p* < 0.001). Our results suggest implementing two familiarization sessions for our HIFT-based workout was sufficient to decrease anxiety and establish a baseline measurement. Future research should examine if this remains true for other types of HIFT-based workouts.

## 1. Introduction

Well-designed research aims to collect high-quality data [1]. However, this can be challenging when working with human subjects. Applied exercise science tries to mitigate one of the challenges of performing research with human subjects (i.e., variability in baseline data) by including familiarization sessions prior to baseline assessments [2]. Indeed, these familiarization sessions are regularly employed in studies that measure maximum aerobic capacity, anaerobic power, muscular strength, power, and endurance [2,3,4,5]. Such familiarization sessions aim to orient subjects to the study protocol by asking them to engage in the exact activity or measure as they will during the study. The familiarization session allows the subjects to learn the protocol, thus, becoming comfortable with the study design in subsequent sessions. Moreover, recent research acknowledges the importance of evaluating the effects of familiarization rather than just stating that familiarizations sessions were held [6,7,8,9].

Another benefit of familiarization sessions is addressing the subject’s anticipatory anxiety response. Anticipatory anxiety is characterized as an unpleasant psychological state in response to feelings of uncertainty and stress concerning the performance of a task [10]. Previous research demonstrates a negative relationship between anticipatory anxiety and performance [10,11,12,13], where physiological, behavioral, and cognitive responses to anxiety may negatively affect motor skills [10,12]. Physiological responses to anticipatory anxiety include increased heart rate and sweating. Indeed, too large of an increase in autonomic arousal is negatively associated with performance [14].

Anxiety may manifest as a component of a subject’s personality (trait anxiety) or a context-dependent temporary experience (state anxiety) [15]. Previous research has used the 40-question State–Trait Anxiety Inventory (STAI) to assess both anxiety aspects [15]. However, the Single Question Anxiety Likert Scale (SQALS) questionnaire, which has proven valid and reliable, is especially useful in measuring state anxiety in repeated measures research [16].

Anticipatory anxiety can also elicit cognitive responses, such as avoidance and negative self-talk, contributing to decreased performance [10,14]. Specifically, positive or negative perceptions of anxiety can mitigate performance due to anticipatory anxiety [17]. These perceptions may become more positive through repeated exposure to an event, thus, reducing anxiety and improving performance.

However, previous research has demonstrated mixed results regarding the importance of a familiarization session in applied exercise science research. For example, increases in strength were shown in repeated one-repetition maximum (1RM) attempts in the bench press, squat, and arm curl within subsequent testing sessions in trained and untrained individuals [18]. Rather than reflecting actual changes in strength, familiarity with the protocol improved the accuracy of 1RM testing. Without multiple baseline attempts, this could have led to conclusions about significant but false pre-to-post-test improvements in strength. Another study suggested that familiarization may be age specific because older women required more familiarization attempts to evaluate strength accurately [19]. Some authors have suggested that familiarization is not required to elicit correct sprint and vertical jump performance [5,20,21]. Indeed, further research is needed to better understand the effect of familiarization on performance, particularly for training programs with high variability in training and testing protocols.

High-intensity functional training (HIFT) is a popular type of exercise that utilizes a variety of workouts that are scaled based on individual strengths and weaknesses to improve fitness outcomes and reduce training volume [22,23,24]. HIFT incorporates aerobic and resistance training at high intensities and emphasizes functional (multi-joint) movements, which have proven effective among varied populations, including HIFT novices, older adults, and military members [25,26,27]. HIFT’s fitness and health benefits have been well documented and include improvements in strength, metabolic conditioning performance, and body composition [28,29,30,31]. Notably, the varied nature of HIFT workouts increases anticipatory anxiety [32] and HIFT practitioners have been found to have moderate levels of both trait and state anxiety [33]. Previous HIFT research demonstrates the necessity for balanced performance testing that is accessible to HIFT novices [34]. Moreover, previous HIFT research has used a single familiarization session for experienced participants prior to testing [35]. Considering HIFT’s physical health benefits, its ability to increase anticipatory anxiety, and its growing popularity, further research may help elucidate the relationship between anxiety, familiarization, and performance and establish an appropriate performance testing protocol for HIFT novices. Therefore, this study aims to assess the effects of familiarization sessions on anticipatory anxiety and HIFT performance. It was hypothesized that familiarization would decrease anticipatory anxiety and improve performance among HIFT novices.

## 2. Materials and Methods

### 2.1. Experimental Approach to the Problem

Subjects attended five sessions and performed four workouts, one workout per session, with at least 48 h of rest between sessions. During the first session, subjects completed anthropometric and body composition assessments. During sessions, 2–5 subjects completed a standardized 10-minute warm-up, followed by a three to five-minute break, then a 10-minute as-many-rounds-as-possible (AMRAP) HIFT workout, described below. All workouts were exactly the same; however, subjects were purposefully uninformed about the workout protocol to facilitate an anticipatory anxiety response. Their anxiety response was measured three times (i.e., upon arrival, after learning of the workout details, and upon completion of the workout) with the SQALS questionnaire. Workout performance was recorded as the total number of repetitions completed at each session. Workout sessions were performed individually in an indoor gym under the direction of a researcher. Subjects were asked not to perform maximum effort exercises 24 h before each workout session.

### 2.2. Subjects

Sixteen healthy, college-aged individuals volunteered to participate in the study (men = 6, women = 10; mean age = 20.2 ± 1.1 years). Subjects were recreationally strength trained, performing at least three strength training sessions per week for at least six months. Subjects had no HIFT experience and were free from injury. Written informed consent was obtained from each subject prior to study commencement. The university’s institutional review board approved the study; approval #10843.

### 2.3. Measurements

#### 2.3.1. Anthropometrics and Body Composition

During the baseline visit, researchers recorded subject height to the nearest 0.1 cm with a Charder stadiometer (Model H.M. 200P; Taichung City, Taiwan). Weight was measured to the nearest 0.1 kg via a Tanita TBF310 bioelectrical impedance scale (Arlington Heights, IL, USA). Body composition was also assessed using the Tanita TBF310 bioelectrical impedance scale.

#### 2.3.2. Single Question Anxiety Likert Scale Questionnaire

This simple, single-item subjective anxiety measure determines an individual’s current (state) anxiety level and is an adequate replacement for the STAI (state) questionnaire [16]. The SQALS questionnaire is particularly useful when collecting repeated measures. Subjects answered the SQALS questionnaire three times at each workout session to measure their anxiety: upon arrival, after they were informed of the workout, and upon workout completion. The SQALS questionnaire was answered online via Qualtrics (Provo, UT, USA). Participants were asked to “choose the number that shows how anxious you feel at the moment”, with answer options ranging from 1 “not at all anxious at the moment”, to 3 “moderately anxious”, to 5 “most anxious you could ever imagine”.

### 2.4. Workout Description

All subjects performed an AMRAP HIFT-style workout of 5 jumping pull-ups, 10 burpees, and 15 air squats for 10 min. Each round had 30 repetitions and the total number of repetitions completed across the 10 min was used as their final performance score. Neither music nor verbal encouragement from researchers was employed to minimize motivational effects. All participants were instructed on how to perform each movement and were required to demonstrate their ability to complete each movement before beginning the workout. The following requirements for completing each movement were consistent across participants and sessions and participants who demonstrated poor form were given verbal correction cues.

- Jumping pull-ups: An overhand grip was taken on the bar, arms fully elongated with the bar’s height at the forearm’s mid-point. The subject was instructed to jump to perform the pull-up and then return to the starting position. The chin had to clear the bar for the repetition to count.
- Burpees: From a standing position, subjects descended to bring their chest and thighs to the ground. On the ascent, subjects returned to standing by jumping or stepping their feet together underneath them, jumping with both feet off the ground and performing an overhead clap.
- Air Squat: From a standing position, subjects lowered their bodies into a squat until their thighs were below parallel to the floor. Subjects fully extended their knees and hips to complete the movement.

### 2.5. Statistical Analysis

Data were analyzed using the R statistical computing environment and language [36] via the Jamovi graphical user interface version 2.2 [37]. Descriptive statistics were calculated and all dependent variables were checked for normality before inference testing. Relationships between the fixed effect (i.e., SQALS and number of trials) and dependent variable (i.e., workout performance) data were assessed using a linear mixed-effects model via the GAMLj: General analysis for linear models Jamovi module [38]. A repeated-measures ANOVA for Jamovi was performed to assess the effect of time on each of the SQALS scores [39]. A Greenhouse–Geisser adjustment was applied for SQALS 2 questionnaire. An alpha level of 0.05 was used for all statistical inferences and post hoc comparisons were adjusted using the Bonferroni correction. All data are reported as mean ± standard deviation.

## 3. Results

**Figure 1:**
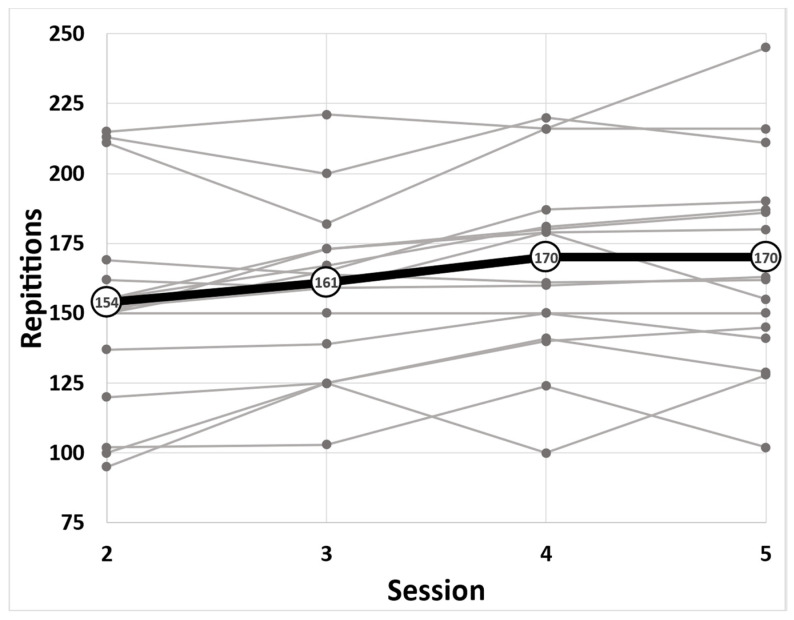
Total repetitions (mean) performed each workout are shown in black. Gray points indicate each individual’s total repetitions for each study session.

Subjects’ anthropometric and body composition data are shown in Table 1. Figure 1 shows each subject’s repetitions and the sample’s mean repetitions in every session. Mean values for anxiety measures are shown in Table 2.

A significant main effect for the number of previous trials on workout performance was observed (*p* = 0.011, *F* = 4.166). An average improvement of 16 additional repetitions was shown in session 4 (*p* = 0.002, *t* = 3.304, 95% CI (6.49, 25.42)) and was sustained for session 5 *(p* = 0.003, *t* = 3.093, 95% CI (5.85, 26.08)) compared to session 2 performance. Performance peaked at session 4 and stabilized for session 5. There was no significant difference in performance between sessions 4 and 5 (mean repetitions = 170, *p* = 1.00, *t* = −0.003). There was no statistically significant relationship between the SQALS 2 questionnaire responses and workout performance (*p* = 0.608) when the random factor (e.g., the individual) is accounted for; however, a large portion of the variance is explained by the random factor (r^2^_conditional_ = 0.9025).

The repeated-measures ANOVA showed a significant main effect of time on SQALS 1 (*p* < 0.001, *F* = 6.71, η^2^ = 0.173). The post hoc test demonstrated a significant difference between session 2 and 5 (*p* = 0.015, *t* = 3.503) as well as between session 3 and 5 (*p* = 0.011, *t* = 3.656). A significant main effect of time was observed for SQALS 2 (*p* < 0.001, *F* = 14.4, η^2^ = 0.374). The post hoc test demonstrated a significant difference between session 2 and 4 (*p* < 0.001, *t* = 4.90), between session 2 and 5 (*p* < 0.001, *t* = 6.33), as well as between session 3 and 5 (*p* = 0.022, *t* = 3.31). A significant main effect of time was observed for SQALS 3 (*p* < 0.001, *F* = 7.03, η^2^ = 0.155). The post hoc test demonstrated a significant difference between session 2 and 4 (*p* = 0.060, *t* = 2.782), between session 2 and 5 (*p* = 0.007, *t* = 3.873) as well as between session 3 and 5 (*p* = 0.018, *t* = 3.416).

## 4. Discussion

This study investigated the effects of familiarization (number of previous trials) on anticipatory anxiety (SQALS questionnaire) and workout performance (repetitions) by measuring anxiety in subjects who performed the same workout four times, despite not knowing the workout details until just prior to completion each time. We hypothesized that increased familiarity with the workout protocol would decrease anxiety and that performance would improve. We were only partially correct as our data suggest that familiarization (number of previous trials) was more important to performance than anxiety, even though anxiety did decrease. Additionally, familiarization alone did not eliminate pre-workout anxiety in HIFT novices. The third time participants completed the workout, their performance significantly improved and was sustained in the final workout session (Figure 1). This indicated that familiarization with the workout improved performance. Our findings also suggest that the strength of the familiarization effect was strongly individual dependent. While overall improvements in workout performance were observed, some subjects were inconsistent from session to session or maintained their initial performance. These inconsistencies suggest that other factors may be present in determining the familiarization effect for each individual

**Figure 2:**
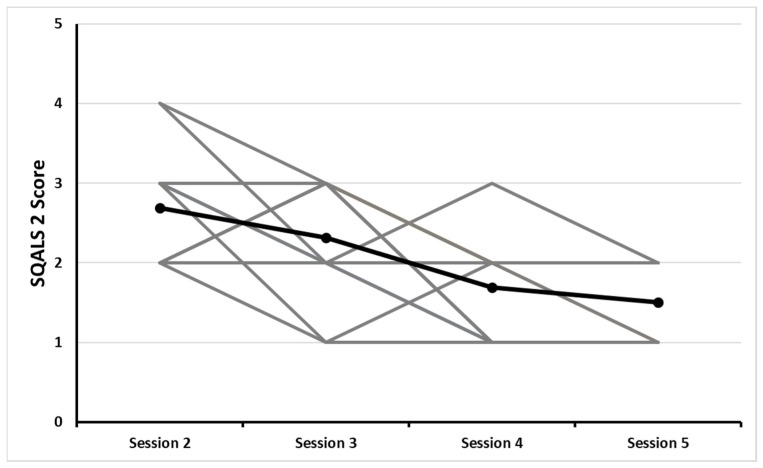
SQALS 2 scores at each workout session. Mean score is shown in black while individual scores are shown in gray.

Anxiety increased from arrival to after learning the workout details during session 2 (i.e., the first time subjects completed the workout). While this increase was not statistically significant, the workout description may have been sufficient to elicit an anticipatory anxiety response. An increase between SQALS 1 and 2 was not observed in subsequent sessions, indicating that familiarization may have played a role in decreasing anticipatory anxiety. During each subsequent session, anxiety was highest at arrival, as subjects expected a new workout, but anxiety lessened during the session after learning the workout was the same as the previous one. Apart from upon arrival at session 3, anxiety decreased at each subsequent session and during each session. However, anxiety was not eliminated. Pre-exercise anticipatory anxiety was insufficient to affect workout performance negatively or positively, thus, contributing inter-individual differences between minimum and maximum SQALS 2 scores. As seen in Figure 2, despite an overall trend of decreasing SQALS 2 scores, some individuals reported an increase or no change in anxiety. Currently, we are unable to explain what contributed to each individual’s level of anxiety.

Our research is similar to previous research that described the need for several familiarization sessions to elicit optimal performance [3]. Amarante de Nascimento et al. [3] tested three strength exercises in trained and untrained women. They found that two to three familiarization sessions were required for precise 1RM attempts to be observed. Our study’s exercise protocol utilized basic movements; however, subjects did not have experience with HIFT. These results suggest that for novices, familiarization with a protocol may be more critical to performance than prior experience. Previous HIFT research using the SQALS questionnaire among experienced HIFT participants demonstrates anxiety peaking before each of two open workouts [32]. However, research employing sprinting and vertical jump protocols only required one familiarization session to observe optimal speed and height [5,20,21]. The exact number of familiarization sessions may be exercise mode and workout dependent.

There is no standardized performance testing protocol for HIFT novices; we attempted to address this deficiency with our study’s workout protocol. Typical testing methods fall into one of three categories: (1) single modality or bioenergetic pathway (e.g., 1RM, VO_2max_), (2) Hero workouts (e.g., Murph: 1-mile run, 100 pull-ups, 200 push-ups, 300 squats, 1 mile run for time with an optional 20 lb weight vest), or (3) benchmark workouts (e.g., Cindy: AMRAP in 20 min of 5 pull-ups, 10 push-ups, 20 squats, Fran: 21-15-9 repetitions of barbell thrusters (men = 95 lb, women = 65 lb) and pull-ups for time) [23]. The benefit of typical HIFT testing is that it is individualized (i.e., can be scaled) based on an individual’s goals and their strengths and weaknesses [24,26]. However, many of the Hero and benchmark workouts require technical skill proficiency, thus, limiting their evaluative quality for novice HIFT subjects like those in our study. Therefore, it is essential that performance testing for HIFT requires multiple body systems to work together in a maximal, balanced, and integrated manner [34]. The workout we used challenged multiple bioenergetic pathways and included movements requiring universal motor patterns (e.g., pulling, pushing, and squatting), making implementation available to all experience levels [27]. However, future research is needed to determine if the number of familiarization sessions needed is consistent across other types of HIFT workouts.

### Strengths and Limitations

Our study has several strengths that should be highlighted. The study protocol was not skill dependent, so individuals at all skill levels could complete the task. We minimized subject burden by implementing a 10-minute, time-efficient protocol and single-question anxiety measurement. Moreover, we used a measurement tool, the SQALS questionnaire, which has been previously used [16].

Nonetheless, several limitations should be addressed. A mask mandate was in effect at the study’s institution and all study subjects were required to wear a mask while exercising. Previous research indicates that wearing a facemask while exercising increases feelings of dyspnea [40]. These dyspnea feelings may have affected the subjects’ anxiety in preparation for and during each workout session. We could not control for student-specific stressors, such as exams, which may have influenced our subjects’ anxiety and performance. Moreover, a larger sample size would be helpful to elucidate further the dynamic interplay between familiarization, anxiety, and workout performance. Lastly, we did not account for changes in diet and supplementation, sleep pattern changes, or the consumption of stimulants prior to study sessions.

## 5. Conclusions

Workout performance improved after two trials and was sustained after three trials, indicating a positive familiarization effect. However, we did not observe a significant relationship between workout performance and anticipatory anxiety. Thus, incorporating two familiarization sessions prior to baseline was necessary for the HIFT-based workout we tested and should be examined for future applied exercise HIFT research. Therefore, to help control for a familiarization effect, researchers should examine the need for multiple familiarization sessions before collecting baseline data. In doing so, researchers give subjects enough time to maximize the familiarization effect and, thus, not interfere with other study variables. This strategy has the potential to improve the data collection quality. Lastly, our data suggest a sizeable individual effect between familiarization and performance. Individual-specific profiling may be required to determine the best protocol for familiarization, as some subjects may experience differing levels of this effect.

## Figures and Tables

**Table 1:** Subject anthropometric and body composition.

Variable	Males (*n* = 6) M ± SD	Females (*n* = 10) M ± SD	Total Sample (*n* = 16) M ± SD
Age (years)	20.7 ± 1.13	19.9 ± 1.06	20.2 ± 1.14
Weight (kg)	82.8 ± 14.20	70.6 ± 9.38	75.2 ± 12.8
Body fat percentage (normal mode)	22.3 ± 4.17	29.6 ± 8.20	26.9 ± 7.78
Body fat percentage (athletic mode)	17.4 ± 4.19	26.4 ± 5.98	23.1 ± 6.91

**Table 2:** Subject anxiety measures scores for each study session.

Anxiety Measure *	Session 2	Session 3	Session 4	Session 5
SQALS 1 (at arrival)	2.38 ± 0.81	2.50 ± 0.82	1.88 ± 0.72	1.63 ± 0.88
SQALS 2 (after learning the workout details)	2.69 ± 0.70	2.31 ± 0.70	1.69 ± 0.60	1.50 ± 0.52
SQALS 3 (after completing the workout)	1.94 ± 0.85	1.63 ± 0.62	1.50 ± 0.63	1.19 ± 0.40

* SQALS is the Single Question Anxiety Likert-Scale (SQALS) question scored between 1 and 5, where a lower score corresponds to lower anxiety.

## Data Availability

Data are available from the corresponding author.
